# A novel approach for segmentation and quantitative analysis of breast calcification in mammograms

**DOI:** 10.3389/fonc.2024.1281885

**Published:** 2024-04-04

**Authors:** Yunfei Tong, Jianrong Jiang, Fang Chen, Guanghua Guo, Chaoren Zhang, Tiana Deng

**Affiliations:** ^1^ Shanghai Yanghe Huajian Artificial Intelligence Technology Co., Ltd., Shanghai, China; ^2^ Mindong Hospital Affiliated to Fujian Medical University, Ningde, Fujian, China

**Keywords:** breast cancer, breast calcification, segmentation, Pro_UNeXt, machine learning

## Abstract

**Background:**

Breast cancer is a major threat to women’s health globally. Early detection of breast cancer is crucial for saving lives. One important early sign is the appearance of breast calcification in mammograms. Accurate segmentation and analysis of calcification can improve diagnosis and prognosis. However, small size and diffuse distribution make calcification prone to oversight.

**Purpose:**

This study aims to develop an efficient approach for segmenting and quantitatively analyzing breast calcification from mammograms. The goal is to assist radiologists in discerning benign versus malignant lesions to guide patient management.

**Methods:**

This study develops a framework for breast calcification segmentation and analysis using mammograms. A Pro_UNeXt algorithm is proposed to accurately segment calcification lesions by enhancing the UNeXt architecture with a microcalcification detection block, fused-MBConv modules, multiple-loss-function training, and data augmentation. Quantitative features are then extracted from the segmented calcification, including morphology, size, density, and spatial distribution. These features are used to train machine learning classifiers to categorize lesions as malignant or benign.

**Results:**

The proposed Pro_UNeXt algorithm achieved superior segmentation performance versus UNet and UNeXt models on both public and private mammogram datasets. It attained a Dice score of 0.823 for microcalcification detection on the public dataset, demonstrating its accuracy for small lesions. For quantitative analysis, the extracted calcification features enabled high malignant/benign classification, with AdaBoost reaching an AUC of 0.97 on the private dataset. The consistent results across datasets validate the representative and discerning capabilities of the proposed features.

**Conclusion:**

This study develops an efficient framework integrating customized segmentation and quantitative analysis of breast calcification. Pro_UNeXt offers precise localization of calcification lesions. Subsequent feature quantification and machine learning classification provide comprehensive malignant/benign assessment. This end-to-end solution can assist clinicians in early diagnosis, treatment planning, and follow-up for breast cancer patients.

## 1 Introduction

**Figure 1:**
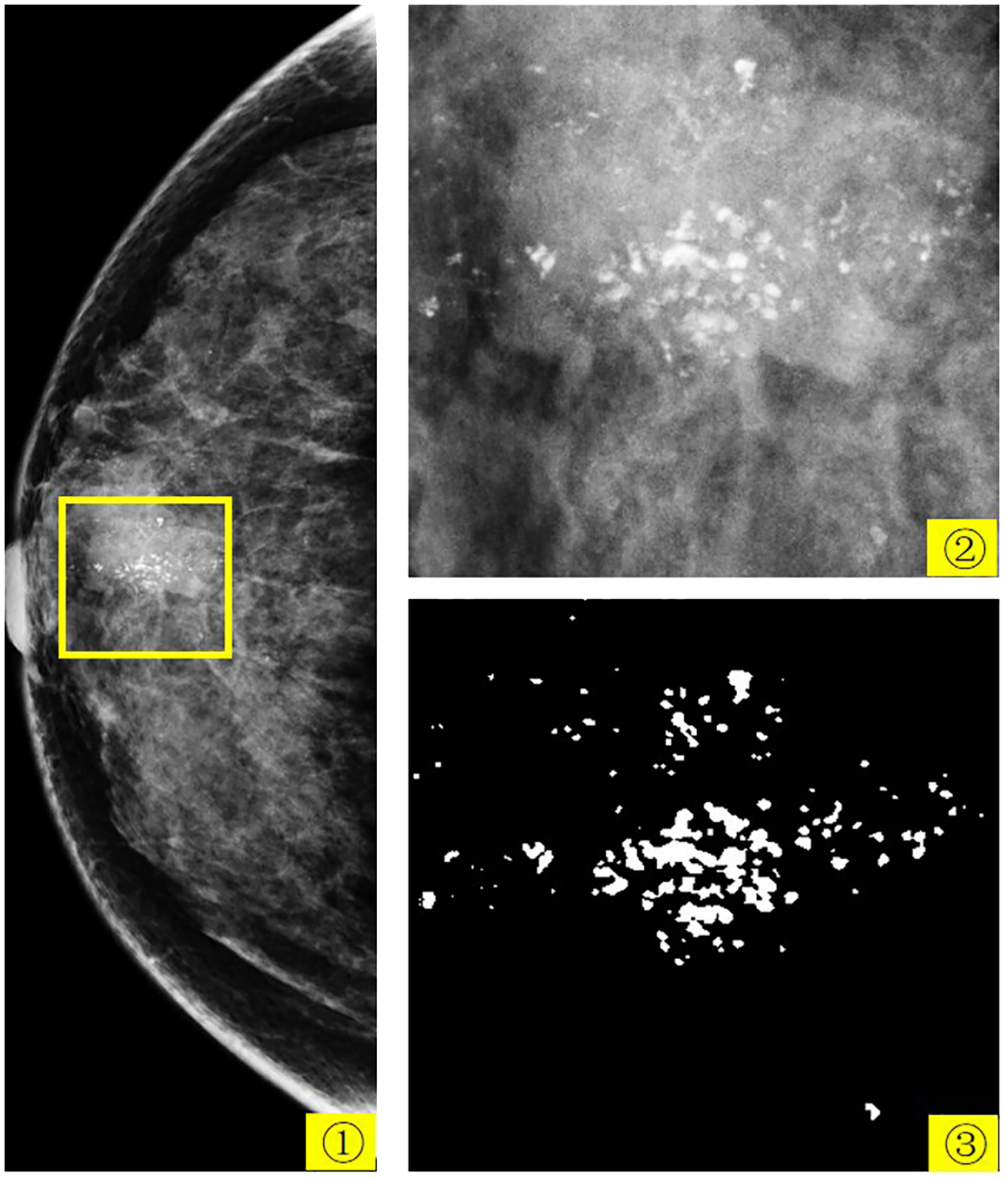
The mammogram of a 57-year-old patient with breast cancer. 1 is the patient’s right CC view, 2 is the yellow box in 1, and 3 is the expert-labeled result of 2.

According to the latest global statistics, breast cancer incidence rates have risen over the last four decades. Breast cancer is the most common cancer (1) and the second leading cause of cancer death among women (2). Mammograms are one of the main methods for detecting and screening breast cancer at an early stage. Although there are many different types of breast lesions in mammograms, calcification cannot be ignored. Calcification refers to the accumulation of calcium deposits within female breast tissue and is an indicator that appears in the initial stages of breast cancer and is often associated with ductal carcinoma *in situ* and invasive cancer. In mammogram, calcification is considered a primary indication of malignancy. In screening programs, between 12.7% and 41.2% of women are recalled for further evaluation due to calcification lesions being the sole sign of potential breast cancer. Analyzing calcification aids in determining the most appropriate approach to patient management. Understanding the morphology, size, and distribution of calcification is crucial in determining whether they are MB (benign and malignant) and whether additional imaging or biopsies are warranted. **Figure 1** shows the mammogram of a 57-year-old patient with breast cancer. In this figure, 1 is the patient’s right craniocaudal (CC) view, 2 is the yellow box within 1, and 3 is the expert-labeled result of 2, where calcification lesions are highlighted as white spots on the mammogram. In breast tissue, calcifications smaller than 0.5 mm are categorized as micro-calcification. While micro-calcification lesions do not always indicate malignancy, their presence often serves as an early warning sign of potential breast cancer.

In recent years, with the development of artificial intelligence technology, the application of artificial intelligence has achieved very good results in many fields (3–6). Computer-aided diagnosis (CAD) in the medical field has also made many breakthroughs based on artificial intelligence, including the brain (7–9), breast (10), thyroid (11), and other parts of the body. CAD algorithms have the potential to offer clinicians improved decision support for early segmentation and analysis of breast calcification. CAD algorithms founded on deep learning have demonstrated their effectiveness and robustness in automating breast cancer analysis. Image segmentation techniques are employed to segment calcification lesions, and among the deep learning-based methods, UNet (12) stands out as an efficient and robust medical image segmentation technique. In recent years, UNet has served as the cornerstone for nearly all leading medical image segmentation methods. Its extensions, such as UNet++(UNetPlus)Zhou et al. (13), V-Net (14), Y-Net (15), and TransUNet (16), have been at the forefront of medical image segmentation.

However, considering that mammograms are typically large-scale images whereas calcification lesions are notably small, especially micro-calcification, the aforementioned methods often face challenges, including an abundance of network parameters, complex computations, and slow processing speeds. In calcification process segmentation, issues like false positives and limited accuracy are prevalent. As a result, some scholars have proposed methods tailored to the unique characteristics of calcification (17–21). Wang and Yang (17) developed a context-sensitive deep neural network designed to simultaneously consider local image features of calcification and the surrounding tissue background for calcification detection. Marasinou et al. (19) proposed a deep learning method based on Hessian matrix Gaussian difference regression, a two-stage multiscale method for calcification segmentation. Valvano et al. (18) proposed a two-stage deep learning method: first, extract region proposals and then classify each region proposal. Hossain (21) proposed a method consisting of multiple preprocessing stages and then manually selected suspicious regions and fed them into a trained UNet network. Zamir et al. (20) developed a strategy to prioritize challenging pixels during the training phase to address the false-positive calcification issue. These methods are generally classified as CAD systems, which automatically flag suspicious calcification lesions in mammograms. While current CAD systems achieve high sensitivity, they also generate numerous false-positive markers, increasing radiologist interpretation time. Most breast calcification CAD systems primarily focus on segmentation or detection of calcification, lacking in-depth analysis of their features. They provide a visual observation but fail to comprehensively analyze and quantify calcification properties. To effectively assess the morphology, size, and distribution of calcification, it is necessary to accurately segment calcification from mammograms. Accurate segmentation allows for a more accurate quantitative description of the morphology, size, and distribution of calcification.

Based on the characteristics of calcification lesions in mammogram and the latest SOTA medical image segmentation algorithm UNeXt (22), this paper proposes a Pro_UNeXt algorithm. Based on the UNeXt algorithm, the Pro_UNeXt algorithm model first adds a micro-calcification learning block at the network input, which can effectively improve micro-calcification segmentation. The fused-MBConv (23) and Tok-MLP modules are used instead of the convolution module in the network, improving the model’s ability to learn features and its operating speed. Based on the characteristics of calcification, the focal loss (24) and Dice loss (25) are used as the loss function in the first step of training, and then the Hausdorff distance (HD) loss (26) is used to fine-tune the model trained in the first step to improve the model’s ability to segment micro-calcification. Due to the difficulty in labeling breast calcification data and that the image area occupied by calcification is very small, effective data augmentation (Aug) methods are used for the characteristics of calcification lesions. These methods include cropping, original image scaling, Gaussian blur, sharpening, grayscale transformation, affine transformation, grayscale histogram transformation (27), and calcification copy and paste (28).

As one of four common breast lesions, calcification is inseparable from breast cancer. To analyze the characteristics of calcification lesions, quantitative analysis is conducted on segmented calcification lesions. First, the quantitative characteristics of each mammogram is quantified. Including the number, density, size, area, distribution, calcification clusters, perimeter, roundness, long side, rectangularity, length–width ratio, and perimeter ratio. Then, the machine learning method is used to classify the MB.

In this paper, the problem of calcification segmentation and quantitative analysis is investigated. The proposed algorithm can not only reduce the calculation overhead and operation cost but also accurately segment micro-calcification. This paper presents a rapid calcification lesion segmentation algorithm that combines deep learning and breast calcification features. At the same time, machine learning methods were used to analyze the quantified calcification lesions. The method can help doctors conduct more effective analyses, assist doctors in film reading, and reduce errors.

## 2 Method

**Figure 2:**
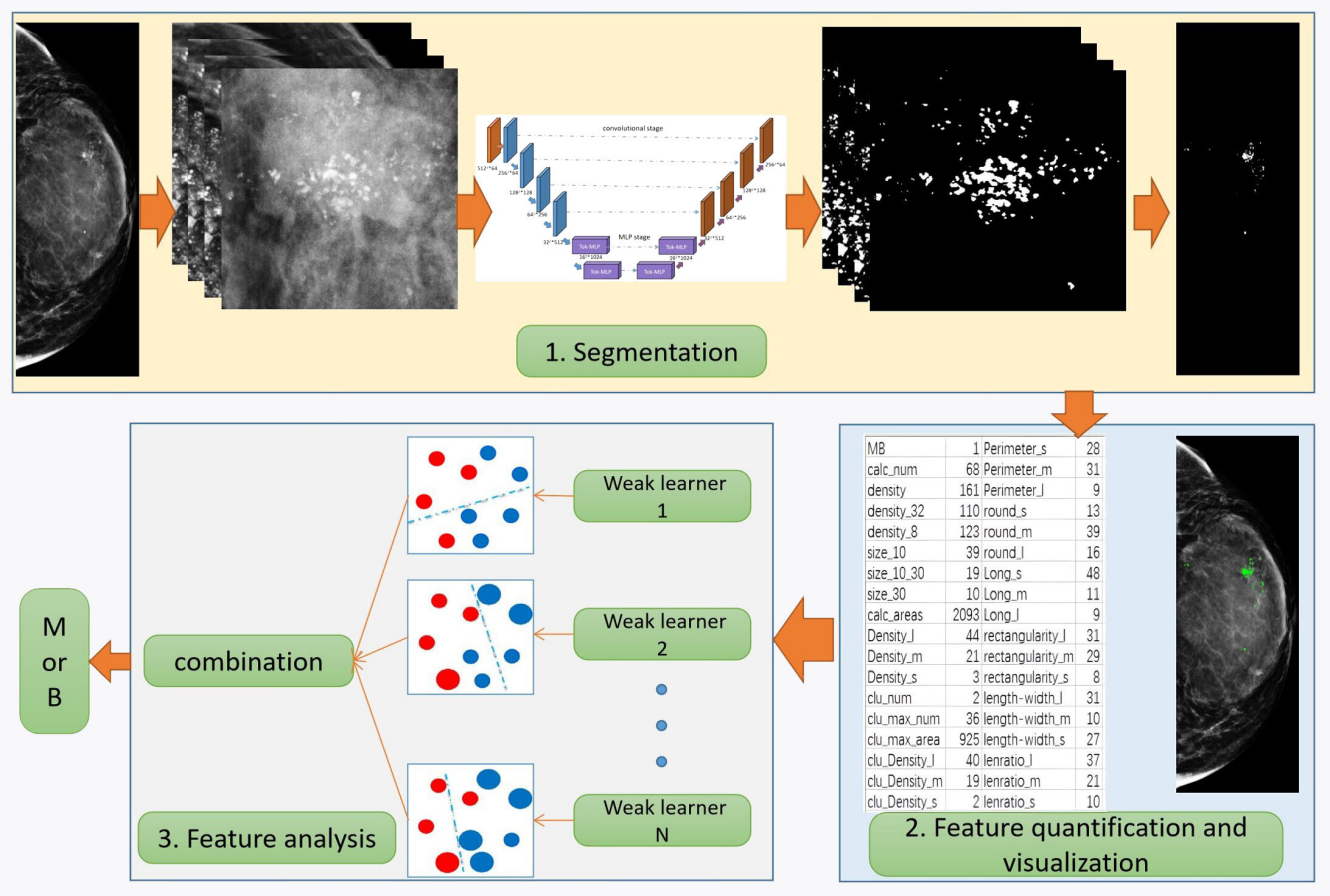
Segmentation and quantitative analysis flowchart of breast calcification in mammograms.

Based on the characteristics of calcification, this paper proposes a novel and comprehensive breast calcification lesion segmentation and analysis scheme. As shown in **Figure 2**, the scheme consists of three modules: segmentation, feature quantification, and feature analysis. The first is the calcification lesion segmentation module. The module designs a Pro_UNeXt algorithm with high accuracy and good performance based on the characteristics of calcification. Next is the calcification lesion feature quantification module and visualization. As one of the most common breast lesions, knowledge of the morphology, size, and distribution of calcification by the radiologist can help determine the extent of the lesion. According to the daily working habits of radiologists, we quantified the characteristics of the calcification segmented in the first step, including quantity, shape, size, density, peripheral density, cluster, and blurriness. The quantification results inform radiologists about various features, reducing the risk of oversight and facilitating analysis comparisons of feature changes. Finally, the calcification feature analysis module employs the machine learning algorithm to classify quantified features, providing a comprehensive assessment of lesion MB.

**Figure 3:**
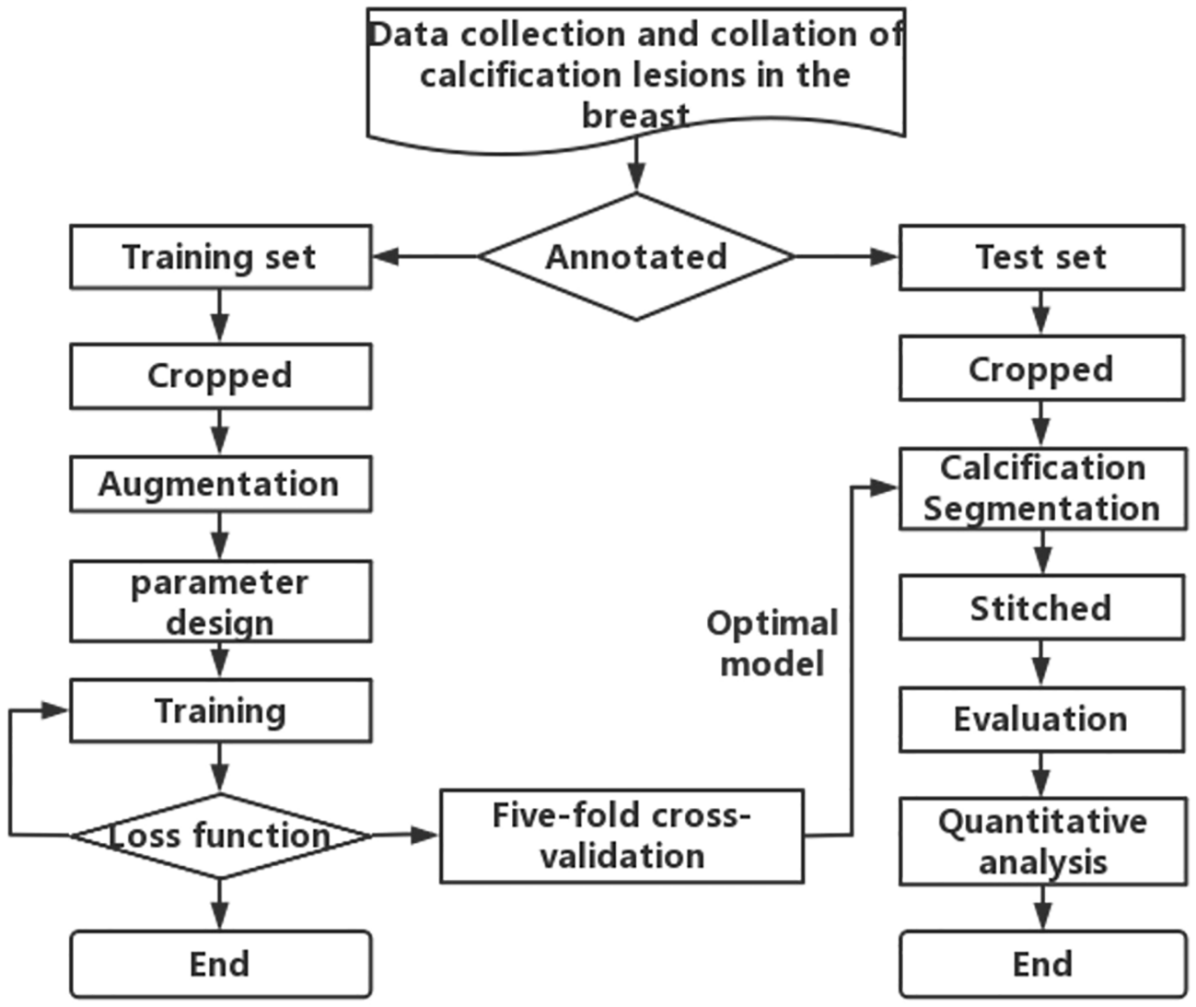
Flowchart for segmentation and quantitative analysis of mammographic calcification lesion.

To obtain the first step of calcification lesion segmentation model, the calcification lesion processing process as shown in **Figure 3** is designed. Mammograms with only calcification lesions were first collected and annotated by two junior radiologists. If the Dice coefficient of the data annotated by junior radiologists was less than 95%, it was considered a discrepancy. If there was discrepancy between the two, it was corrected by a senior radiologist. The datasets involved in this article were all annotated and checked by radiologists. The annotated data were divided into a training set and a test set at the ratio of 4:1. Since breast calcification lesions are localized, the training and test sets were cropped to the size of 512 × 512 pixels. To reduce calcification on the edges of image, the overlap rate between cropped images was 20%. As shown on the left side of **Figure 3**, the model training process includes cropped, Aug, model parameter design, model training, loss function calculation, and fivefold cross-validation. The optimal models for the five validation sets were obtained through loop iterations, with each model being trained for 200 iterations. As shown on the right of **Figure 3**, the test set after cropping was segmented with five train models. The segmentation results were stitched together to obtain a map of calcification lesions for each mammogram. The Dice, specificity (SPE), recall, and intersection over union (IoU) (29) of the models were calculated to evaluate the segmentation models. Finally, the segmentation results were quantified and analyzed. To highlight the superior calcification segmentation performance of the algorithm in this study, comparable algorithms were employed using the same dataset and procedures. This was done to ensure the effectiveness of the algorithm. The annotation method employed in this article involves utilizing the labeling functions within the 3D Slicer software (30), specifically the level tracing feature found in the Segment Editor module. This entails setting constraints on the density range of calcifications and then clicking on the center of the calcification, thereby enabling the acquisition of the boundary of the calcified region.

### 2.1 Data Aug

According to the calcification characteristics, the Aug methods were as follows: cropping, original image scaling, Gaussian blurring, sharpening, grayscale transformation, affine transformation, grayscale histogram transformation, and copy and paste. In this paper, the original image was directly scaled and then cropped, which can improve the micro-calcification detection ability of the model. Image blurring and grayscale transformation can improve the segmentation of high fibrous density and fuzzy micro-calcification lesions. The copy and paste strategy is based on copying the calcification points in an image, counting the number of connected domains, selecting the connected domains using a random ratio (0 to 1), and then pasting them into other images. This method not only increases the number of annotated calcification points but also enhances the performance of model.

### 2.2 UNeXt algorithm

The UNeXt algorithm is a convolutional multilayer perceptron (MLP) (31)-based image segmentation network. It is designed with an early convolution stage and a latent-stage MLP stage. The early convolution stage is responsible for extracting low-level features from the input image, whereas the latent-stage MLP stage is used for modeling the representations.

One of the key components of UNeXt is the tokenized MLP (32) block. This block effectively tokenizes and projects the convolutional features and uses MLPs to model the representations. The tokenization process involves dividing the input into non-overlapping patches and flattening each patch into a 1D vector. These vectors are then projected into a higher dimensional space using a linear transformation. The projected vectors are then fed into the MLPs for further processing. UNeXt changes the channel of the input when entering the MLP to focus on learning local dependencies and improving the segmentation ability of object edges. Using tokenized MLP in the latent space not only reduces the number of parameters and computational complexity but also produces better representations to help segmentation. To further enhance the performance, channel shuffling is proposed before feeding the input into the MLPs. This allows the network to focus on learning local dependencies, which are crucial for image segmentation tasks.

Similar to UNet, the network also includes skip connections between the encoder and decoder at all levels. Compared with the current medical image segmentation architecture, UNeXt has 72 times fewer parameters, 68 times less computational complexity, and 10 times faster inference, while achieving better segmentation performance than state-of-the-art medical image segmentation architectures. This makes UNeXt a promising solution for real-time, fast image segmentation tasks in clinical applications.

### 2.3 Pro UNeXt algorithm

**Figure 4:**
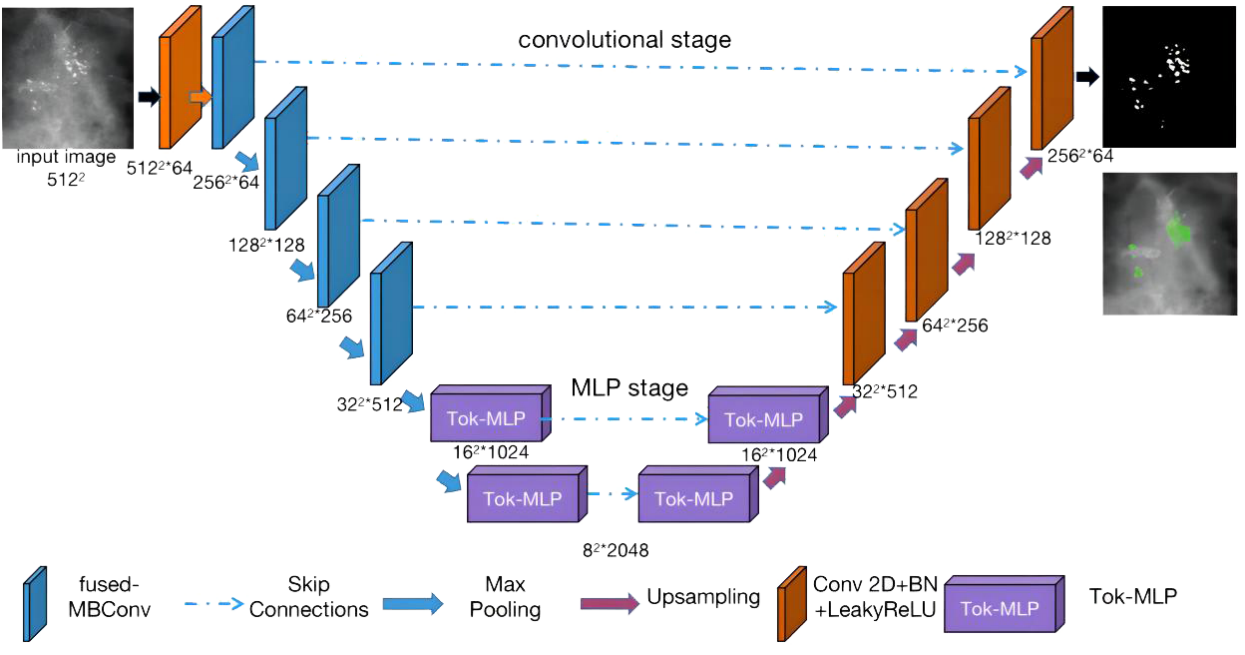
Image segmentation algorithm Pro_UNeXt network structure.

Combining the characteristics of breast calcification lesions, this paper proposes the Pro_UNeXt algorithm based on UNeXt. The Pro_UNeXt medical image segmentation is shown in **Figure 4**. Our algorithm adopts a UNet architecture consisting of an encoder and a decoder. The encoder comprises one convolutional block (orange), four fused-MBConv (23) blocks (blue), and two tokenized MLP blocks (purple). The decoder contains two tokenized MLP blocks (purple) and four convolutional blocks (orange).

In the encoder part, the first convolution block(orange) is not downsampled to achieve maximum retention of the micro-calcification features. To learn micro-calcification features, each image is fed into the first convolution block with 64 channels and a step of 1. After the first convolutional block (orange), the next 4 blocks of the network are fused-MBConv blocks (blue). To learn image features more accurately, the convolutional kernel in the encoding process is fused-MBConv. Fused-MBConv, compared with traditional convolutions, excels in parameter efficiency, feature adaptability with Squeeze-and-Excitation (SE) (33) blocks, reduced computational load, and suitability for resource-constrained environments. Its efficiency and versatility make it valuable in deep learning models, leading to improved accuracy in computer vision tasks, particularly in mobile and embedded applications. The last two blocks are tokenized MLP blocks (purple). The tokenized MLP block efficiently tokenizes and projects convolutional features into a higher-dimensional space, enabling the network to capture complex patterns. It focuses on local dependencies and reduces parameters and computational complexity, making the network more efficient for real-time applications.

The decoder part contains two tokenized MLP blocks (purple) and four convolutional blocks (orange) from bottom to top. In addition to the first micro-calcification convolutional block (orange), each other encoder block reduces the feature resolution by 1/2, and each decoder block increases the feature resolution by a factor of 2.

The other parameters of our algorithm are as follows: the input image size is fixed at 512 × 512, where the numbers of parameters in each channel layer of the encoding process are 64, 64, 128, 256, 512, 1,024, and 2,048 and the numbers of decoding parameters are 2,048, 1,024, 512, 256, 128, and 64. The network structure is an encoder–decoder architecture with two phases: (1) convolution phase and (2) tokenized MLP phase.

Convolution phase: This phase comprises both normal convolution blocks (orange) and fused-MBConv blocks (blue). Each normal convolution block includes a convolutional layer, a batch normalization layer, and a ReLU activation layer. The convolutional layers employ a kernel size of 3 × 3 with a stride of 1 and padding of 1. The fused-MBConv convolutional block comprises a 1 × 1 convolutional layer, a Squeeze-and-Excitation (SE) layer, and 3 × 3 convolutional layers. Within the encoder, the convolution block incorporates a maximum pooling layer with a pooling window of 2 × 2. Conversely, the convolution block within the decoder is composed of transposed convolutions. These convolutions act as learnable upsampling modules, enhancing the capacity for learnable parameters and improving boundary detection.

Tokenized MLP phase: First, the features are shifted and projected into tokens. Then, these tokenized blocks are passed to the shifted MLP (across the width). Next, the features are passed through the depthwise convolutional (DWConv) (34) layer. DWConv is used for two reasons: (1) It helps encode the positional information of the MLP features. (2) It uses fewer parameters compared with regular convolutions. The layer is then activated using the Gaussian Error Linear Unit (GELU) (35), which is a smoother alternative to the Rectified Linear Unit (ReLU) and has better performance. Afterward, the features are passed through another moving MLP (across height), and the original token blocks are connected using a residual structure. Finally, the application layer is normalized, and the output features are passed to the next block.

In summary, our Pro_UNeXt algorithm has three advantages: (1) The use of a micro-calcification learning block at the input to focus on detecting small lesions. (2) Replacing standard convolution blocks with fused-MBConv and Tok-MLP to improve feature learning and speed. (3) Increasing network channels for enhanced representation capacity.

### 2.4 Loss function

In deep learning, the loss function is used to measure the gap between the model’s prediction results and the real results. Our goal is to minimize the loss so that the model’s predictions become more accurate. In this paper, three loss functions are used to train the model: focal loss, Dice loss, and HD loss. The focal loss is a loss function that handles imbalanced sample issues. The Dice loss is a metric used to evaluate the similarity of two samples and is currently widely used in medical image segmentation. The HD loss is a boundary-based metric commonly used to segment small target. However, when the HD loss is used alone to train a neural network, training instability may occur. Therefore, in this paper, the focal loss + Dice loss combination is first used as the loss function for initial training, and then the HD loss is used to fine-tune the trained model to obtain better performance.

### 2.5 Statistical analysis method

**Figure 5:**
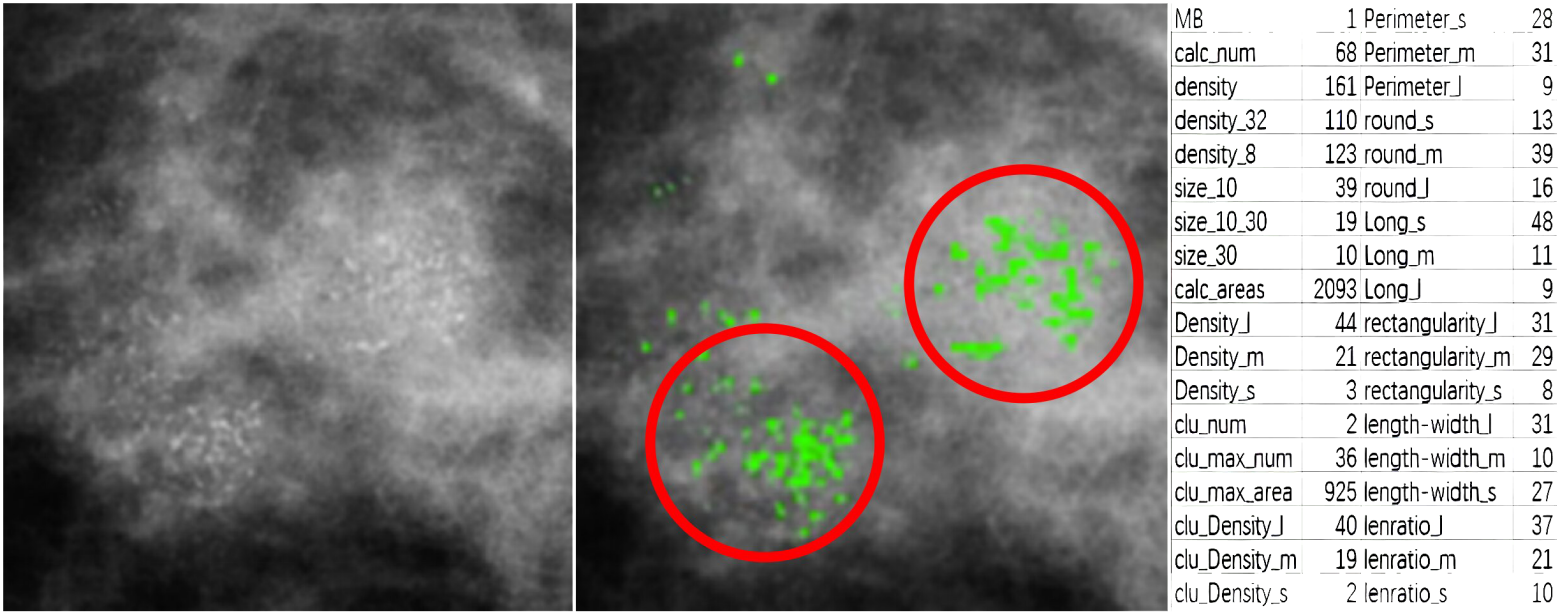
A partial interception of mammographic calcification (left), calcification annotations (middle), and quantitative features table of calcification(right).

For feature analysis of medical image segmentation, radiomics is most commonly used. However, radiomics is not applicable for breast calcification lesions. Therefore, in this paper, other methods were used to learn the characteristics of calcification lesions. **Figure 5** shows a partial interception of a mammographic calcification image (left), the calcification annotations (middle), and a table of quantitative features for the calcification (right). The MB in the statistical results is provided by pathological analyses. The calc_num indicates the number of calcification. The density represents the average density of calcification. The density_32 is the average density within a 32-pixel field surrounding each calcification. The density_8 is the average density within an 8-pixel field. The size_10 is the number of calcification less than 10 pixels. The size_10_30 is the number of calcification with sizes between 10 and 30 pixels. The size_30 is the number with sizes above 30 pixels. The calc_area indicates the total area of calcification. The suffixes _l, _m, and _s represent relative size categories. For example, density_l, density_m, and density_s indicate high-, medium-, and low-density calcification respectively. length_l, length_m, and length_s denote calcification with high, medium, and low aspect ratios. The clu_num, clu_max_num, and clu_max areas represent the number of clusters, number of calcification in the largest cluster, and area of the largest cluster. clu_Density_l, clu_Density_m, and clu_Density_s denote the number of high-, medium-, and low-density calcification within clusters. perimeter_s, perimeter_m, and perimeter_l indicate calcification with small, medium, and large perimeters. round_s, round_m, and round_l represent different roundness levels. long_l, long_m, and long_s denote different lengths of the long side of approximated rectangles. rectangularity_l, rectangularity_m, and rectangularity_s represent different rectangularity levels. lenratio_l, lenratio_m, and lenratio_s indicate different perimeter ratios. The statistical method for identifying calcification clusters is as follows: First, a dilation operator with a 16 × 16 all-one matrix is applied on the segmented image. This connects adjacent calcification points into connected domains. If the number of calcification points in a connected domain is greater than 5, it is considered a calcification cluster. As shown in **Figure 5**, two calcification clusters are highlighted within the red circles.

To help doctors accurately judge patients’ MB, machine learning algorithms are used to analyze quantitative features, including decision tree (DTs) (36), logistic regression (LR) (37), support vector machines (SVMs) (38), K nearest neighbors (KNN) (39), random forests (RFs) (40), XGBoost (XGB) (41), and AdaBoost (42). The relationship between quantitative features and MB is established by the machine learning algorithm. It can provide more accurate digital services for clinics and help doctors make more accurate judgements. It is also of great significance for retrospective comparisons of patient follow-up.

### 2.6 Evaluation method

For the evaluation of calcification segmentation, the Dice (**Equation 1**), recall (**Equation 3**), SPE (**Equation 2**) and IoU (**Equation 4**) are used. These metrics are commonly employed in the evaluation of machine learning models, particularly in image segmentation. They provide valuable insights into different aspects of a model’s performance, such as its ability to correctly identify positive and negative instances, and the degree of overlap between predicted and true positive regions. The formula is as follows:


(1) Dice= 2TP/(2TP+FP+FN)

(2) SPE = TN/(TN + FP)

(3) Recall = TP/(TP + FN)

(4) $$\text{IoU }=\;\frac{\text{TP}}{\text{TP }+\text{ FP }+\text{ FN}}$$

where TP is the true positive, TN is the true negative, FP is the false positive and FN is the false negative.

The 95% confidence interval provides a range of plausible values for a parameter estimate, conveying the level of uncertainty associated with the estimate. The p-value helps assess the strength of evidence against a null hypothesis in hypothesis testing, guiding the decision to accept or reject the null hypothesis based on a predetermined significance level (commonly 0.05). The ROC curve is a graphical representation used to evaluate the performance of a binary classifier. It is a plot of the TP rate against the FP rate at different classification thresholds. The AUC is the area under the ROC curve and is used as a metric to measure the performance of a binary classifier. It ranges from 0 to 1. The higher the AUC is, the better the classifier performance.

## 3 Image segmentation results

### 3.1 Experimental conditions and the dataset

To train and test our model, we used one public dataset and one private dataset. The DDSM (43) is a public dataset created by medical institutions in the United States. We manually screen DDSM data with calcification lesions. A total of 401 benign and 358 malignant cases with calcification lesions were collected in the public dataset. The private dataset was provided by Mindong Hospital Affiliated with Fujian Medical University. As shown in **Table 1**, there were a total of 178 cases of mammograms, including 76 cases of malignant (average age 50.5) and 102 cases of benign (average age 47.9). The benign and malignant are generated by histopathology. The table includes age, breast density, and BI-RADS distribution. Their P values are 0.18, 0.27, and 0.69, respectively. It shows that there is no significant difference between benign and malignant data. Due to certain differences between the two datasets, both datasets were independently tested and trained, without cross-validation between them.

**Table 1:** General clinical characteristics of the private dataset.

Characteristics	Total	Malignant	Benign	P value
Mean age	49.0	50.5	47.9	
Age range	23-85	27-85	23-67	0.18
Breast density				0.27
Almost entirely fatty	8 (4.5%)	2 (2.6%)	6 (5.9%)	
Scattered densities	21 (11.2%)	7 (9.2%)	14 (13.7%)	
Heterogeneous dense	131 (73.6%)	56 (73.7%)	75 (73.5%)	
Extremely dense	18 (10.1%)	11 (14.5%)	7 (6.9%)	
BI-RADS classification				0.69
II	35 (19.7%)	0 (0%)	35 (34.3%)	
III	29 (16.3%)	5 (6.6%)	24 (23.5%)	
IVa	48 (27.0%)	11 (14.5%)	37 (36.3%)	
IVb	26 (14.6%)	20 (26.3%)	6 (5.9%)	
IVc	23 (12.9%)	23 (30.3%)	0(0%)	
V	17 (9.5%)	17 (22.4%)	0(0%)	
Total	178	76	102	

The parameters of our machine are Windows 10 Gen Intel(R) Core(TM) i7-11700K @ 3.60 GHz, 32 GB of memory, and 16 GB of graphics card A4000 memory. The private dataset acquisition machine is a GE full digital mammogram machine, which can display micro-calcification lesions less than 0.1 mm, and the single pixel is 0.068 mm. The data annotation software used was 3D slicer (30).

### 3.2 Image segmentation results

To verify the effectiveness of the segmentation algorithm, two datasets are used for testing: DDSM and a private dataset. Our algorithm is compared with classical algorithms: UNet, UNetPlus, and UNeXt, where UNet and UNetPlus use ResNet (44) and EfficientNet (45), respectively, as the backbone. The models of the comparison algorithm and Pro UNeXt+Aug+Loss are the result of optimization with Aug and loss functions.

#### 3.2.1 DDSM dataset results

**Figure 6:**
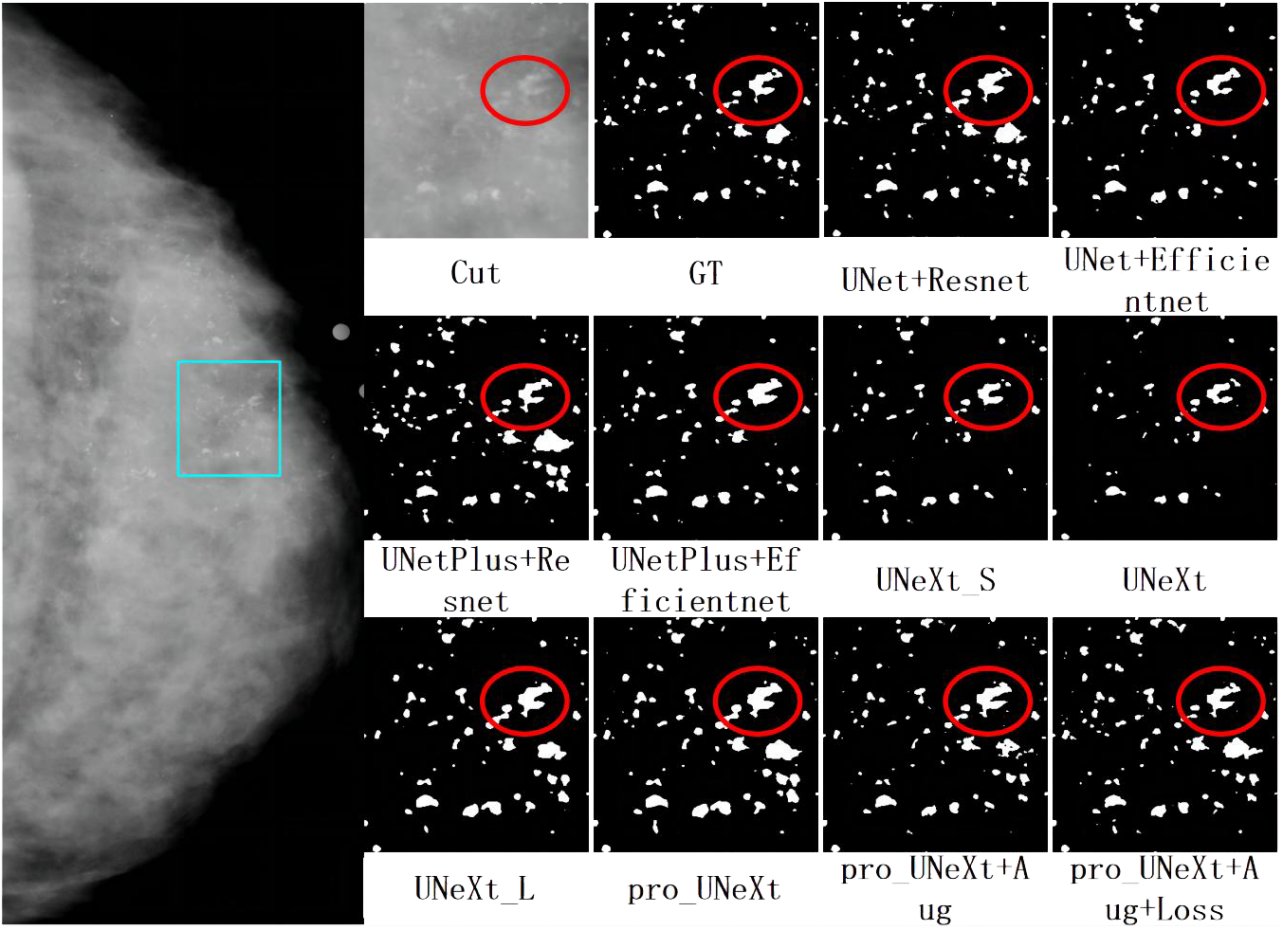
The first on the left is the CC view of the patient, where the area in the light blue box is compared for segmentation effect. Others are cut image, GT, and segmentation results of different algorithms.

As shown in **Figure 6**, we selected the CC view image of the patient P-01108 in the DDSM, and the pathological result was malignant. The area in the light blue box is compared for different segmentation algorithms. Cut is the cropping image of the light blue box, and GT is the binary image of the cropping area marked by the imaging doctor. Others are segmentation results of different algorithms. the Dice scores of different segmentation algorithms in this image were as follows: UNet+ResNet: 0.77; UNet+EfficientNet: 0.80; UNetPlus+ResNet: 0.78; UNetPlus+EfficientNet: 0.76; UNeXt_S: 0.57; P UNeXt: 0.76; UNeXt_L: 0.75; Pro_UNeXt: 0.76; Pro_UNeXt+Aug: 0.82; and Pro_UNeXt+Aug+Loss: 0.94. GT is the ground truth of the manual annotation. By observing the red circles, we found that the UNeXt series of algorithms missed a lot of micro-calcification lesions. However, Pro_UNeXt, which adds a micro-calcification learning block, has a stronger detection ability for micro-calcification and muddy calcification. Compared with the UNet series algorithm, the Pro_UNeXt algorithm performs better in details.

As shown in **Table 2**, we tested different algorithms on the DDSM dataset. After the optimization with Aug and loss functions, Pro_UNeXt+Aug+Loss achieved the highest Dice score of 0.823 (95% CI 0.747–0.901). Compared with the algorithm without Aug and loss function optimization, the Dice score increased from 0.794 (95% CI: 0.703–0.865) to 0.823 (95% CI: 0.747–0.901). The Dice score of Pro_UNeXt+Aug+Loss is 0.060 higher than that of the UNeXt series algorithms and 0.006 higher than that of the UNet series algorithms. Regarding other metrics, the Pro_UNeXt algorithms also showed excellent performance and obtained the optimal Dice of 0.823, IoU of 0.665, and SPE of 0.999. The recall is 0.01 lower than that of the best UNet+EfficientNet algorithm. The Pro_UNeXt algorithm only requires 7 ms to process a single 512 × 512 image, which is 3 ms slower than the original UNeXt. Compared with the speeds of the UNet and UNetPlus algorithms, the speed of Pro_UNeXt is more superior. Since the area occupied by calcification is very small relative to the entire image, the SPE of all algorithms is close to 1. After comparison, it is not difficult to see that our algorithm not only has better speed but also has the best performance in calcification segmentation.

**Table 2:** The segmentation results of different algorithms tested in the DDSM dataset.

Model	Dice (95%CI)	IoU	SPE	Recall	T(ms)
UNet+ResNet	0.786 (0.673-0.895)	0.597	0.998	0.809	14
UNet+EfficientNet	0.817 (0.706-0.892)	0.642	0.998	**0.837**	19
UNetPlus+ResNet	0.788 (0.687-0.861)	0.549	**0.999**	0.819	20
UNetPlus+EfficientNet	0.809 (0.734-0.866)	0.647	**0.999**	0.772	27
UNeXt_S	0.763 (0.699-0.841)	0.581	**0.999**	0.743	**3**
UNeXt	0.749 (0.657-0.817)	0.559	0.998	0.806	4
UNeXt_L	0.728 (0.603-0.811)	0.557	**0.999**	0.717	19
Pro_UNeXt	0.794 (0.703-0.865)	0.626	0.998	0.802	7
Pro_UNeXt+Aug	0.817 (0.723-0.896)	0.644	**0.999**	0.813	7
Pro_UNeXt+Aug+Loss	**0.823 (0.747-0.901)**	**0.665**	**0.999**	0.827	7

Bold numbers represent the optimal metric results.

#### 3.2.2 Private dataset results

**Figure 7:**
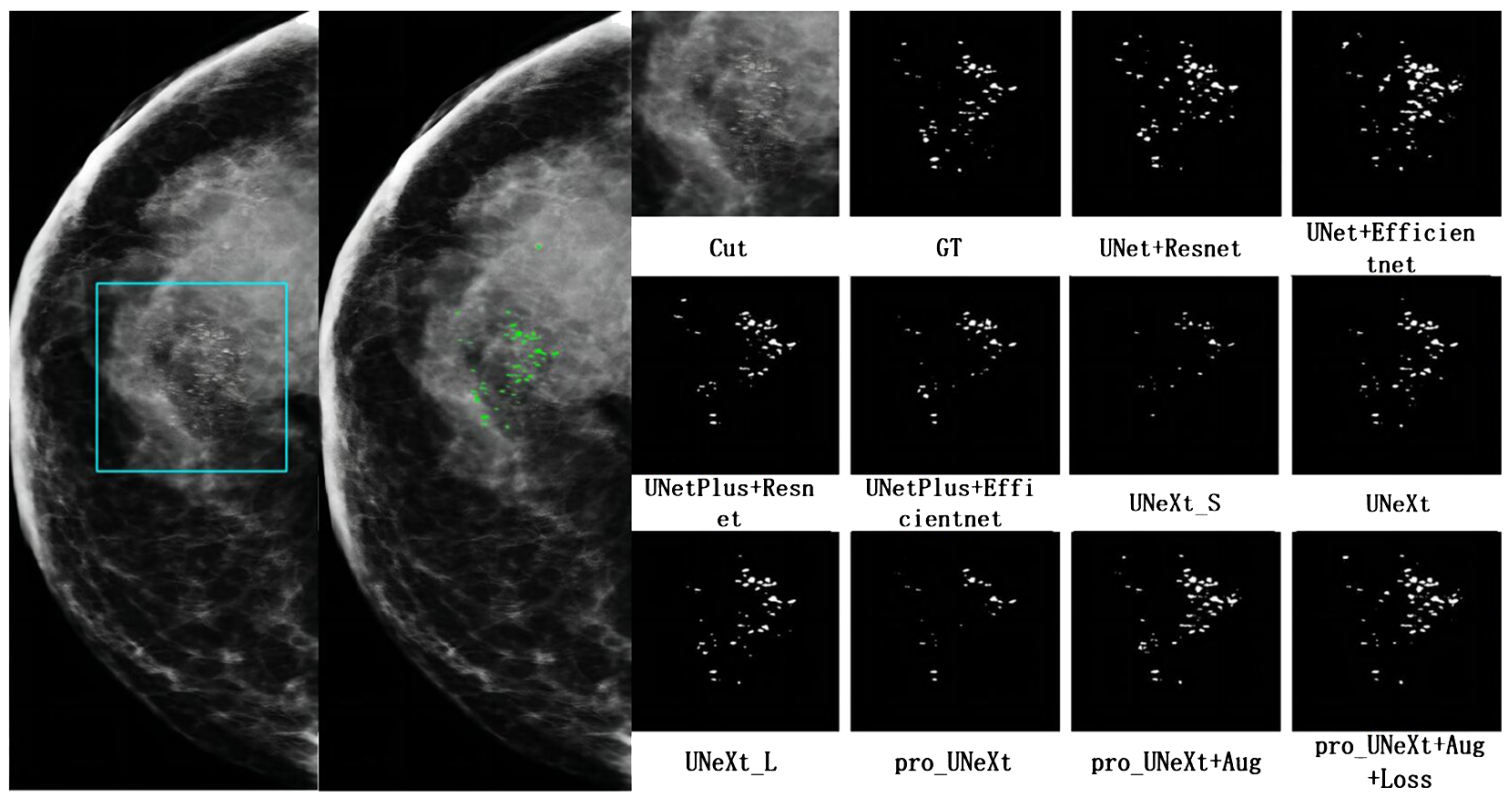
The mammogram of a breast cancer patient in the private dataset. The first on the left is the result of the pro_UNeXt+Aug+Loss, where green is the segmented calcification point. The second on the left is the CC view of the patient, where the area in the light blue box is compared for segmentation effect. Others are cut image, GT, and segmentation results of different algorithms.

**Figure 8:**
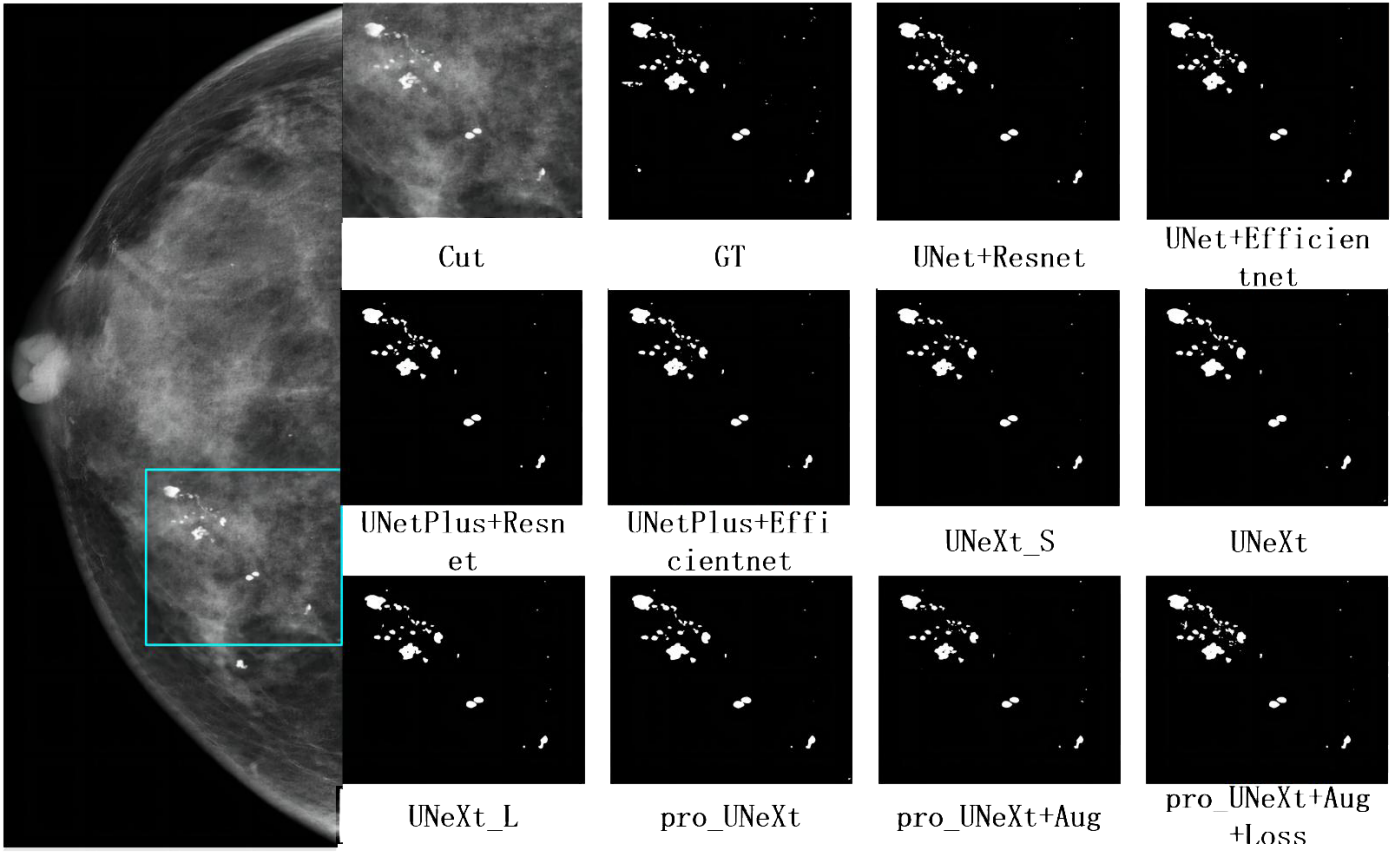
The mammogram of a patient with benign lesions. The first on the left is the CC view of the patient, where the area in the light blue box is compared for segmentation effect. Others are cut image, GT, and segmentation results of different algorithms.

**Figure 7** shows a malignant case in private dataset containing only calcification lesions with indistinct margins. The first on the left is the CC view of the patient, where the area in the light blue box is compared for segmentation. The second on the left is the result of the pro_UNeXt+Aug+Loss, where green is the segmented calcification point. Others are Cut image, GT, and segmentation results of different algorithms. As shown in **Figure 7**, our algorithm has better performance in terms of both missed segmentation and edges, especially micro-calcification. **Figure 8** presents a benign case in the Private dataset with macro-calcification. For this type of macro-calcification, our algorithm achieved the highest Dice score of 0.921, with most algorithms attaining Dice scores in the range of 0.88 to 0.91. From the two figures, we can see that in the private data set, our algorithm has better segmentation of muddy calcification, micro-calcification, and macro-calcification and has almost no false positives.

We conducted experiments on the test set in private dataset. Segmentation performance is presented in **Table 3**. The Pro UNeXt algorithm achieved a Dice score of 0.811 (95% CI 0.737–0.892), outperforming other methods. After optimization with Aug and loss functions, the Pro UNeXt+Aug+Loss model improved the Dice score to 0.838 (95% CI 0.769–0.911). The Pro_UNeXt algorithm was 0.057 better than the UNeXt series algorithm. It was also 0.028 better than the UNet series algorithm. Additionally, Pro_UNeXt demonstrated superior IoU, specificity, and recall versus all other segmentation algorithms.

**Table 3:** The segmentation results of different algorithms tested in private dataset.

Model	Dice (95%CI)	IoU	SPE	Recall
UNet+ResNet	0.779 (0.704-0.871)	0.603	**0.999**	0.747
UNet+EfficientNet	0.810 (0.736-0.878)	0.632	**0.999**	0.790
UNetPlus+ResNet	0.780 (0.702-0.834)	0.605	**0.999**	0.742
UNetPlus+EfficientNet	0.798 (0.718-0.877)	0.627	**0.999**	0.735
UNeXt_S	0.772 (0.710-0.852)	0.581	**0.999**	0.716
UNeXt	0.781 (0.715-0.876)	0.595	**0.999**	0.746
UNeXt_L	0.766 (0.709-0.834)	0.585	**0.999**	0.704
Pro_UNeXt	0.811 (0.737-0.892)	0.638	**0.999**	0.758
Pro_UNeXt+Aug	0.824 (0.753-0.910)	0.643	**0.999**	0.791
Pro_UNeXt+Aug+Loss	**0.838 (0.769-0.911)**	**0.670**	**0.999**	0.795

Bold numbers represent the optimal metric results.

In this section, we selected three typical case images from two data sets and compared the results of different segmentation algorithms. The Pro_UNeXt algorithm can accurately segment both micro-calcification and macro-calcification. In terms of performance, the Pro_UNeXt algorithm of optimization with Aug and loss functions, customized for breast calcification lesions, attained the best results on both the DDSM and private datasets. This confirms its capability in detecting small lesions like micro-calcification. Despite the performance gains, Pro_UNeXt retained excellent computational efficiency. Relative to UNet+EfficientNet (19 ms), it reduced the runtime by over 60% to 7 ms per image.

## 4 Data analysis

Breast calcification segmentation can effectively solve the problem of difficulty in detecting calcification. However, breast calcification has the characteristics of diffuse distribution, small size, blurred edges, and uneven density. Therefore, imaging doctors still need to make subjective judgments after segmentation. This subjective and experience-dependent judgment method can easily lead to missed diagnosis and misdiagnosis. Therefore, this article will perform statistical analysis on two datasets after segmentation. To analyze the characteristics, the segmented images were divided into training and test sets at a 4:1 ratio. Then, quantitative features are calculated for each segmented image, and machine learning is used to classify the features into benign and malignant. The algorithms assessed were DT, LR, SVM, KNN, RF, XGB, and AdaBoost. After training, model performance was evaluated on the test set.

**Figure 9:**
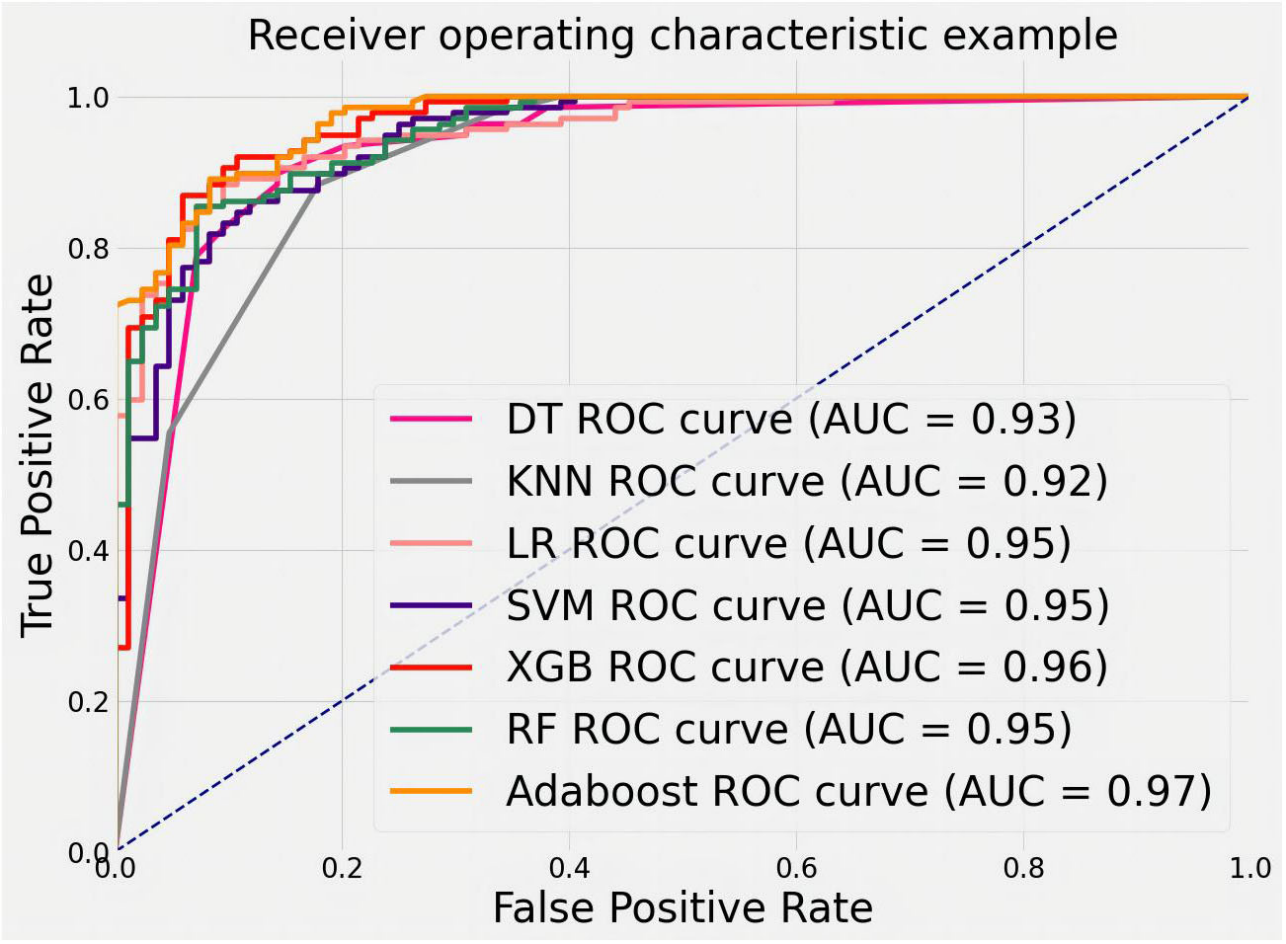
ROC curves for feature classification of private datasets by different machine learning algorithms.

**Figure 10:**
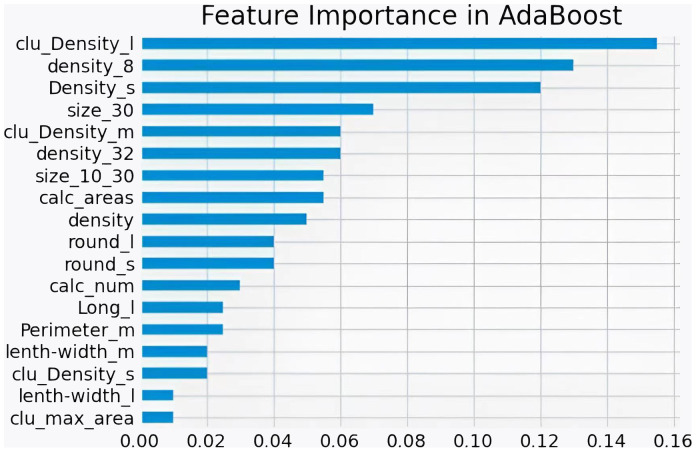
Feature Importance of AdaBoost in private datasets.

**Figure 9** shows the ROC curves of different machine learning algorithms for MB classification in the private dataset. The AUC values, 95% CI, and accuracy rates of the algorithms were as follows: DT’s AUC = 0.93 (95% CI: 0.90–0.97, accuracy rate of 90%), KNN’s AUC = 0.92 (95% CI: 0.89–0.97, accuracy rate of 87%), LR’s AUC = 0.95 (95% CI: 0.93–0.98, accuracy rate of 89%), SVM’s AUC = .95 (95% CI: 0.92–0.98, accuracy rate of 91%), XGB’s AUC = 0.96 (95% CI: 0.94–0.98, accuracy rate of 91%), RF’s AUC = 0.95 (95% CI: 0.93–0.98, accuracy rate of 90%), and AdaBoost’s AUC = 0.97 (95% CI: 0.96–0.99, accuracy rate of 93%). To further understand the importance of each feature, we performed feature importance analysis on the AdaBoost algorithm model. The results are shown in **Figure 10**, which indicates that the density of calcification clusters, calcification density, size, and the density around calcification lesions play a significant role in distinguishing between benign and malignant cases. This is consistent with radiologists’ daily assessment of calcification. Combined with the algorithm’s AUC of 0.97, it directly demonstrates the effectiveness of our quantified features in distinguishing MB cases.

**Figure 11:**
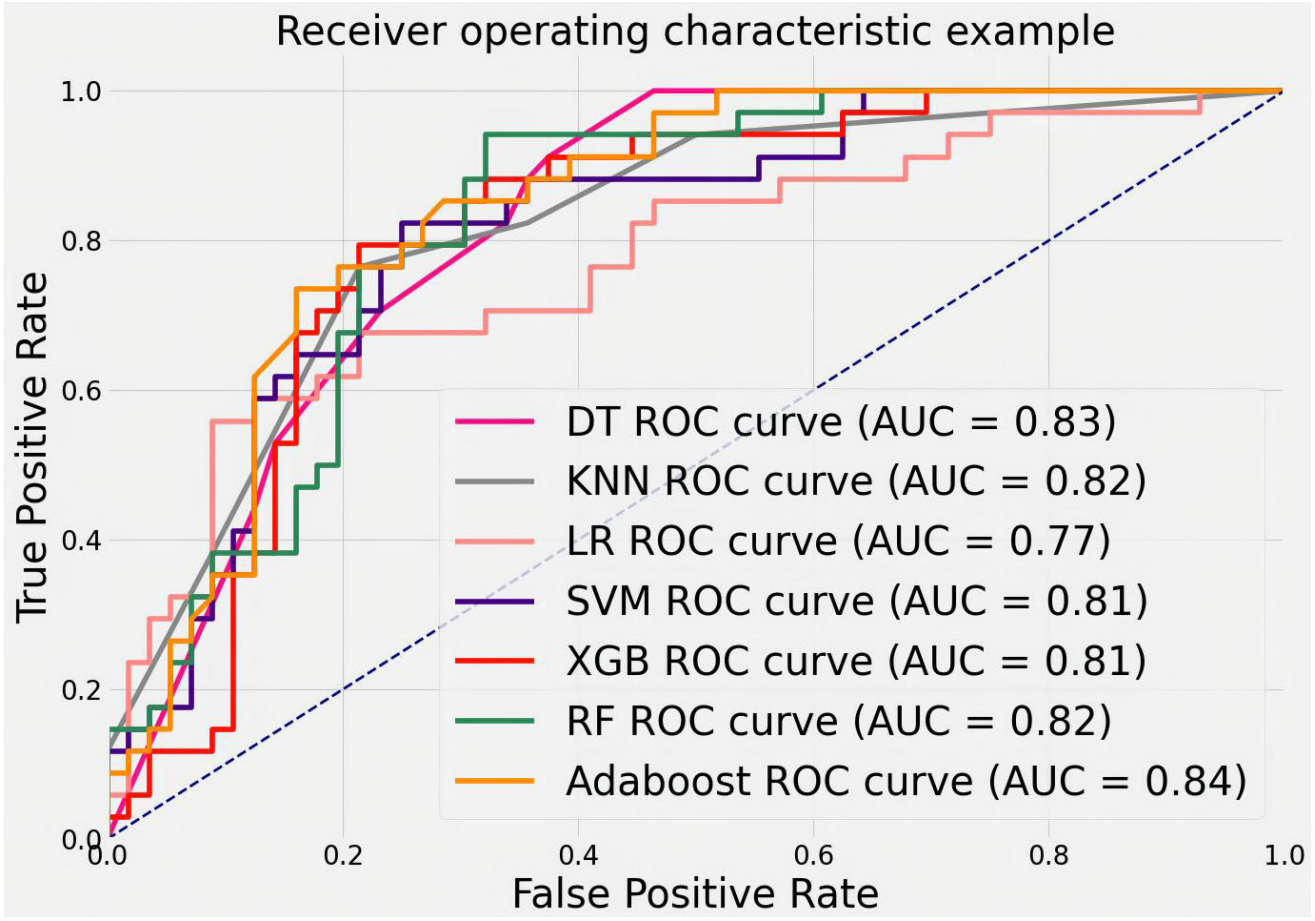
ROC curves for classifying features in the DDSM public dataset using various machine learning algorithms.

To evaluate the ability of features to judge MB, we trained and tested with the public dataset DDSM. The results are shown in **Figure 11**. The AUCs values, 95% CI, and accuracy rates of the algorithms were as follows: DT’s AUC = 0.83(95% CI: 0.73–0.90, accuracy rate of 78%), KNN’s AUC = 0.82(95% CI: 0.74–0.90, accuracy rate of 79%), LR’s AUC = 0.77(95% CI: 0.70–0.85, accuracy rate of 71%), SVM’s AUC = 0.81(95% CI: 0.72–0.90, accuracy rate of 79%), XGB’s AUC = 0.81(95% CI: 0.74–0.90, accuracy rate of 76%), RF’s AUC = 0.82(95% CI: 0.75–0.91, accuracy rate of 78%), and AdaBoost’s AUC = 0.84(95% CI: 0.76–0.91, accuracy rate of 79%). Most classifiers achieved AUCs above 0.80, with AdaBoost again showing the top performance. The consistent results validate that the proposed quantitative features are representative and highly useful for identifying MB.

In this section, our study statistically analyzes two datasets post-segmentation, dividing images into training and test sets (4:1 ratio). Quantitative features are calculated, and machine learning (DT, LR, SVM, KNN, RF, XGB, AdaBoost) classifies them as benign or malignant. AdaBoost exhibits superior performance (AUC = 0.97), emphasizing the importance of features like calcification cluster density and size in distinguishing cases. From **Table 1**, it can be seen that 21.1% of data in malignant cases are classified as below IVa, whereas 42.2% of data in benign cases are classified as above IVa. More than 33.1% cannot accurately determine the condition. Our algorithm’s AUC is above 80%; evaluation on a public dataset (DDSM) and the private dataset confirms the accuracy of proposed features and the efficacy of quantified features in identifying malignant breast calcifications.

## 5 Summary

Breast cancer is the most prevalent malignancy in women globally. Assessing and quantifying breast calcification can inform optimal patient management, thereby improving survival and quality of life. However, the obscurity of calcification renders them prone to oversight, depriving patients of timely treatment. To address this, a technique for detecting breast calcification lesions is needed. Building on calcification characteristics and the state-of-the-art UNeXt segmentation algorithm, this study proposes the Pro UNeXt algorithm. The key innovation are: (1) an improved UNeXt segmentation network using convolutional multilayer perceptron architecture. Pro_UNeXt first adds a micro-calcification learning block at input to improve segmentation performance. Fused-MBConv and Tok-MLP modules replace original convolution modules to boost feature learning and speed. (2) The Pro_UNeXt training process, with focal loss + Dice loss as the initial loss function, followed by fine-tuning with Hausdorff distance loss to refine microcalcification segmentation. (3) Effective data augmentation strategies to reinforce microcalcification segmentation. Experiments demonstrate Pro_UNeXt achieves superior performance on the DDSM and private datasets based on breast calcification lesions. It delivers highly accurate calcification segmentation result at 7 ms per image.

To analyze the characteristics of calcification, the characteristics were quantified for each calcification lesion after segmentation. MB classification of lesions was achieved using machine learning methods. The machine learning algorithms included DT, LR, SVM, KNN, RF, XGB, and AdaBoost. After training, different models were derived from these algorithms. With the private dataset, AdaBoost achieved an AUC of 0.97, demonstrating its superior analytical capability. For the public DDSM dataset, the AUC of AdaBoost was 0.84. Compared with other methods, AdaBoost consistently delivered the best classification accuracy on both datasets. These results validate that the quantitative features proposed in this study are representative and highly discerning for MB identification.

Although the Pro UNeXt algorithm conducts comprehensive analysis of calcification, the private dataset only contained 178 cases. The selective use of calcification lesions likely introduced biases, which may explain the lower accuracy on the private dataset. Additionally, calcification is just one of the four major breast lesions. The sole focus on calcification in this study presents limitations for real-world usage. Therefore, incorporating other types of lesions for holistic analysis will be necessary to better serve clinical needs and improve diagnostic performance. Next, we will carry out the following three tasks: 1. Combined with other lesion characteristics, further analyze the benign and malignant lesions, Bi-Rads grading and molecular classification. 2. Add multi-modal data for joint analysis, including DBT, MLO, and CC, clinical information, etc., to improve model performance. 3. Combined with the follow-up data, compare and analyze the changes in calcification to study the changing characteristics of different types of breast calcification.

In summary, this paper proposes a novel and comprehensive breast calcification lesion segmentation and analysis scheme, consisting of three modules: segmentation, feature quantification, and feature analysis. The first is the calcification lesion segmentation module. The module designs a Pro_UNeXt algorithm with high accuracy and good performance based on the characteristics of calcification. Compared with other calcification segmentation algorithms, the Pro_UNeXt algorithm can quickly and accurately segment calcification lesions, reduce false positives, improve diagnostic efficiency, and reduce the diagnostic time of radiologists. Next is the calcification lesion feature quantification module. To better describe breast calcification, this paper introduces a method for quantifying calcification features, including quantity, shape, size, density, peripheral density, cluster, and blurriness. The quantification results effectively inform radiologists about various features, reducing the risk of oversight and facilitating post-analysis comparisons of feature changes. Finally, the calcification feature analysis module employs the AdaBoost machine learning algorithm to classify quantified features, providing a comprehensive assessment of lesion MB. The algorithm exhibits excellent classification accuracy on two datasets, demonstrating the precision of our quantified calcification features in assessing calcification MB. This capability aids radiologists in making more informed decisions and planning for patient care. The proposed scheme offers a comprehensive solution for breast calcification segmentation and quantitative analysis. It enhances radiologists’ ability to visually inspect lesions, perform more effective analyses, and efficiently interpret mammographic images, ultimately reducing diagnostic errors. Patients benefit from follow-up and retrospective comparisons of calcification lesions. The solution has the potential to alleviate radiologists’ workload, decrease misdiagnosis rates, and improve the quality of life for breast cancer patients, with significant clinical application value.

## Data availability statement

The data analyzed in this study are subject to the following license and access restrictions: Restriction: The datasets are proprietary and contain sensitive information, which necessitates controlled access to ensure confidentiality and compliance with privacy regulations. Researchers interested in accessing these datasets should submit a formal request outlining their research objectives, methodology, and intended use of the data. Requests must adhere to institutional review board (IRB) guidelines and any applicable data protection laws. Interested parties should direct their requests, along with the required documentation, to Dr. JJ at 13509572826@163.com.

## Author contributions

YT: Writing – original draft, Writing – review & editing. JJ: Writing – original draft, Writing – review & editing. FC: Writing – review & editing. GG: Writing – review & editing. CZ: Writing – review & editing. TD: Writing – review & editing.
